# IMPACT OF HIGH SCHOOL BACKGROUND ON OLD AGE COGNITION: EVIDENCE FROM A WISCONSIN COHORT

**DOI:** 10.1093/geroni/igae098.1356

**Published:** 2024-12-31

**Authors:** Michael Topping, Michal Engelman

**Affiliations:** University of Wisconsin Madison, Madison, Wisconsin, United States; University of Wisconsin Madison, Madison, Wisconsin, United States

## Abstract

Individuals with higher academic performance in high school, those who participated in enriching activities, and those with higher socioeconomic status are known to have cognitive advantages in later life. Yet, less is known about whether and how these factors constitute unique social typologies of students in high school. Such background typologies may confer differing cognitive benefits and may have different consequences for later life cognition. Using unique longitudinal survey and cognitive assessment data from the Wisconsin Longitudinal Study and latent class analysis, we identify five different typologies of high school students in 1957: high disadvantage, moderate disadvantage, low advantage, moderate advantage and high advantage. We then estimated regression models examining differences in immediate recall, delayed recall, and letter fluency across different timepoints (2004, 2011, and 2020). We find that compared to the most disadvantaged, those from other class backgrounds see higher average scores for immediate recall and letter fluency as they enter old age in 2004, and maintain these advantages through to 2011. This effect dissipates for some classes in 2020 when WLS participants were nearing the oldest-old ages (80+). Class differences in delayed recall were more clearly apparent in 2011 than either before or after. In addition to socioeconomic mobility and changes in cognition over the life course, these results also reflect health and mortality-related changes to the composition of the cohort at successively older ages.

